# THE EFFECTS OF SOCIAL NETWORK ON RESILIENCE OF COMMUNITY-DWELLING OLDER ADULTS LIVING ALONE

**DOI:** 10.1093/geroni/igz038.3313

**Published:** 2019-11-08

**Authors:** Sangmi Park, Tae Hui Kim, Soyeon Choi, Kyuwon Lee, Jisoo Jung, Myounghee Hong

**Affiliations:** 1 Wonju Severance Christian Hospital, Wonju, Korea, Republic of; 2 Wonju Severance Christian Hospital, Wonju, Gangwon, Korea, Republic of

## Abstract

Resilience is one of the components for successful aging and is related to wellbeing in late life. Studies have shown that older people living alone have low resilience. However, most of these studies were mainly conducted on unhealthy participants. The aim of this study is to examine the factors that contribute to resilience of healthy older adults living alone. Older people living alone who are not subject to public health care service provided to the economically or physically challenged or depressed people were recruited. Data collected from 295 participants were used to conduct hierarchical multiple regression analyses, controlling demographic characteristics, level of cognitive and physical functions, and emotional status. A self-reported questionnaire, UCLA Loneliness Scale, Lubben Social Network Scale(LSNS), and Multidimensional Individual and Interpersonal Resilience Measure(MIIRM) were used to measure study variables. A hierarchical model accounted for 48.8% of the variance in resilience. In model 1(demographics), the religion(β=.178, p<.001) and the perceived economic status(β=-.176, p<.001) variables were significantly related to resilience. The subjective health(β=-.109, p=.038) in model 2(level of function) and the loneliness(β=-.379, p<.001) in model 3(emotional status) had a significant effect on resilience. In model 4, the size(β=-.115, p=.029) and the frequency(β=.160, p=.003) of social networks significantly predicted resilience. The results showed that protecting older adults’ social networks could lead to promote their health and wellbeing. What can be inferred from this finding is that even though the members are small, the social network they often have contact with is important for the resilience of older adults living alone.

